# The genome sequence of the Sulphur Tubic, *Esperia sulphurella* (Fabricius, 1775)

**DOI:** 10.12688/wellcomeopenres.19212.1

**Published:** 2023-04-06

**Authors:** Peter W.H. Holland, University of Oxford and Wytham Woods Genome Acquisition Lab, Darwin Tree of Life Barcoding collective, Wellcome Sanger Institute Tree of Life programme, Wellcome Sanger Institute Scientific Operations: DNA Pipelines collective, Tree of Life Core Informatics collective, Darwin Tree of Life Consortium

**Affiliations:** 1 Department of Biology, University of Oxford, Oxford, England, UK

**Keywords:** Esperia sulphurella, Sulphur Tubic, genome sequence, chromosomal, Lepidoptera

## Abstract

We present a genome assembly from an individual male *Esperia sulphurella* (the Sulphur Tubic; Arthropoda; Insecta; Lepidoptera; Oecophoridae). The genome sequence is 453.2 megabases in span. Most of the assembly is scaffolded into 30 chromosomal pseudomolecules, including the assembled Z sex chromosome. The mitochondrial genome has also been assembled and is 16.2 kilobases in length.

## Species taxonomy

Eukaryota; Metazoa; Ecdysozoa; Arthropoda; Hexapoda; Insecta; Pterygota; Neoptera; Endopterygota; Lepidoptera; Glossata; Ditrysia; Gelechioidea; Oecophoridae; Oecophorinae; *Esperia*; *Esperia sulphurella* (Fabricius, 1775) (NCBI:txid2870497).

## Background

*Esperia sulphurella* is a small moth in the family Oecophoridae sometimes called the ‘Sulphur Tubic’. Although primarily a day-flying species, the adults will also come to light at night. In the UK the moth is widely distributed but probably under-recorded due to its small size (12–16 mm wingspan). The moth is also common in the Netherlands and Belgium and there are scattered records from across Europe, plus some records from the west coast of the United States where it is likely an imported species ( GBIF Secretariat, 2022; Powell, 1968; Powell & Opler, 1996). *E. sulphurella* can be common at some woodland and garden sites in southern England and Wales where dead wood is present, and the adults can be seen in April and May in sunny patches ( NBN Atlas, 2022). The moth has distinctive wing markings comprising a rich brown ground colour flecked with bright yellow scales, many of which are grouped to form a triangle at the trailing edge of each forewing forming a diamond shape when viewed from above. Females also have a pronounced yellow streak along the wing ( Asher, 2013).

The moth has one generation per year, with the adults laying eggs in crevices on bark. The larvae live in silken tubes inside dry rotting wood, including dead trunks of coniferous and deciduous trees, stacked wood piles and fence posts, but never close to where the wood is touching wet ground ( Harper *et al.*, 2002). The larvae may eat rotting wood and associated fungi.

A genome sequence of *E. sulphurella* will be useful in analyses of molecular adaptations to feeding on wood and fungus, and as part of wider comparative studies into genome evolution in the Lepidoptera.

### Genome sequence report

**Figure 1.**
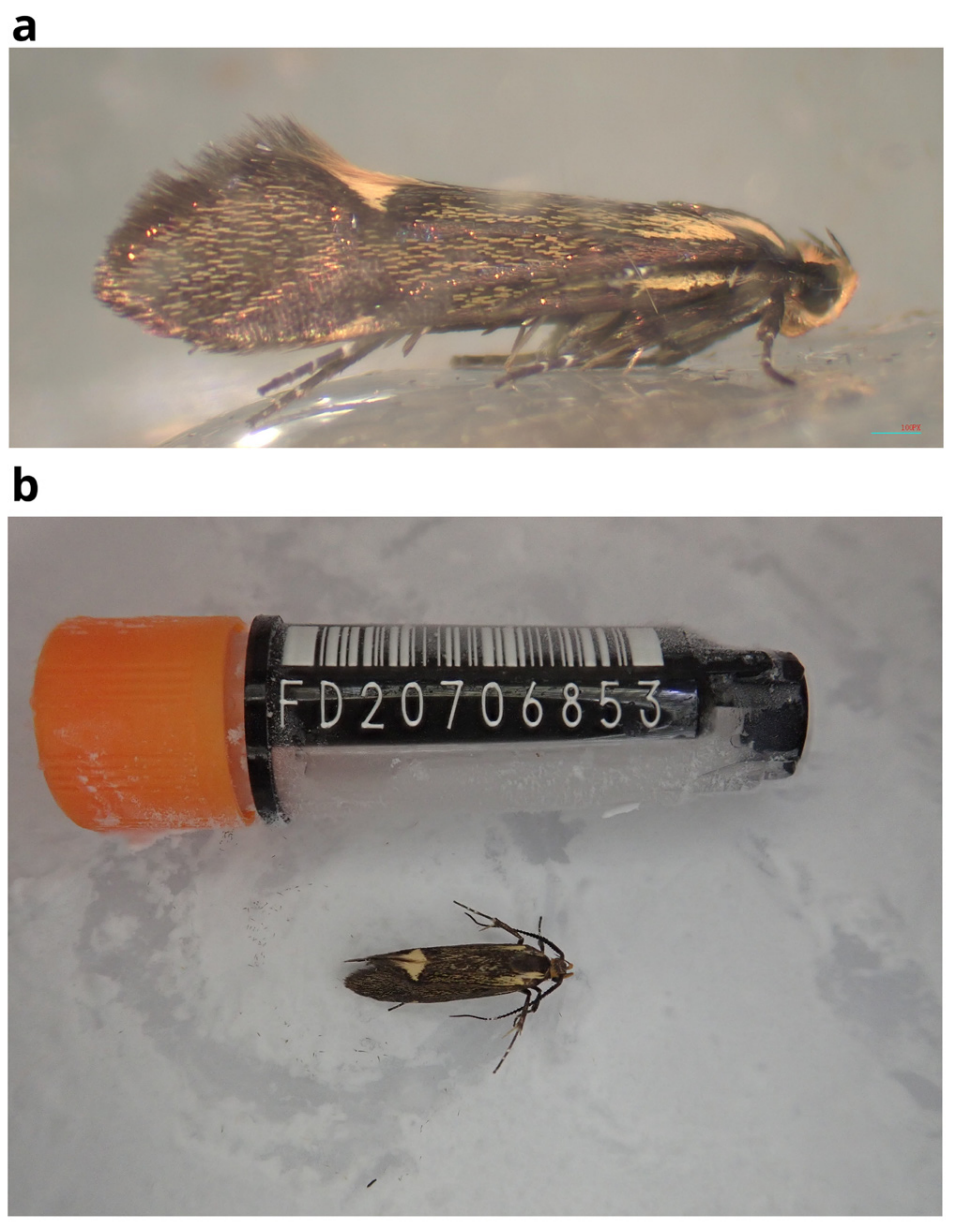
Photographs of the *Esperia sulphurella* (ilEspSulp1) specimen used for genome sequencing.

The genome was sequenced from one male *Esperia sulphurella* ( Figure 1) collected from Wallingford, Oxfordshire (latitude 51.60, longitude –1.14). A total of 62-fold coverage in Pacific Biosciences single-molecule HiFi long reads was generated. Primary assembly contigs were scaffolded with chromosome conformation Hi-C data. Manual assembly curation corrected 22 missing or mis-joins and removed six haplotypic duplications, reducing the assembly length by 0.21% and the scaffold number by 12.5%.

**Figure 2.**
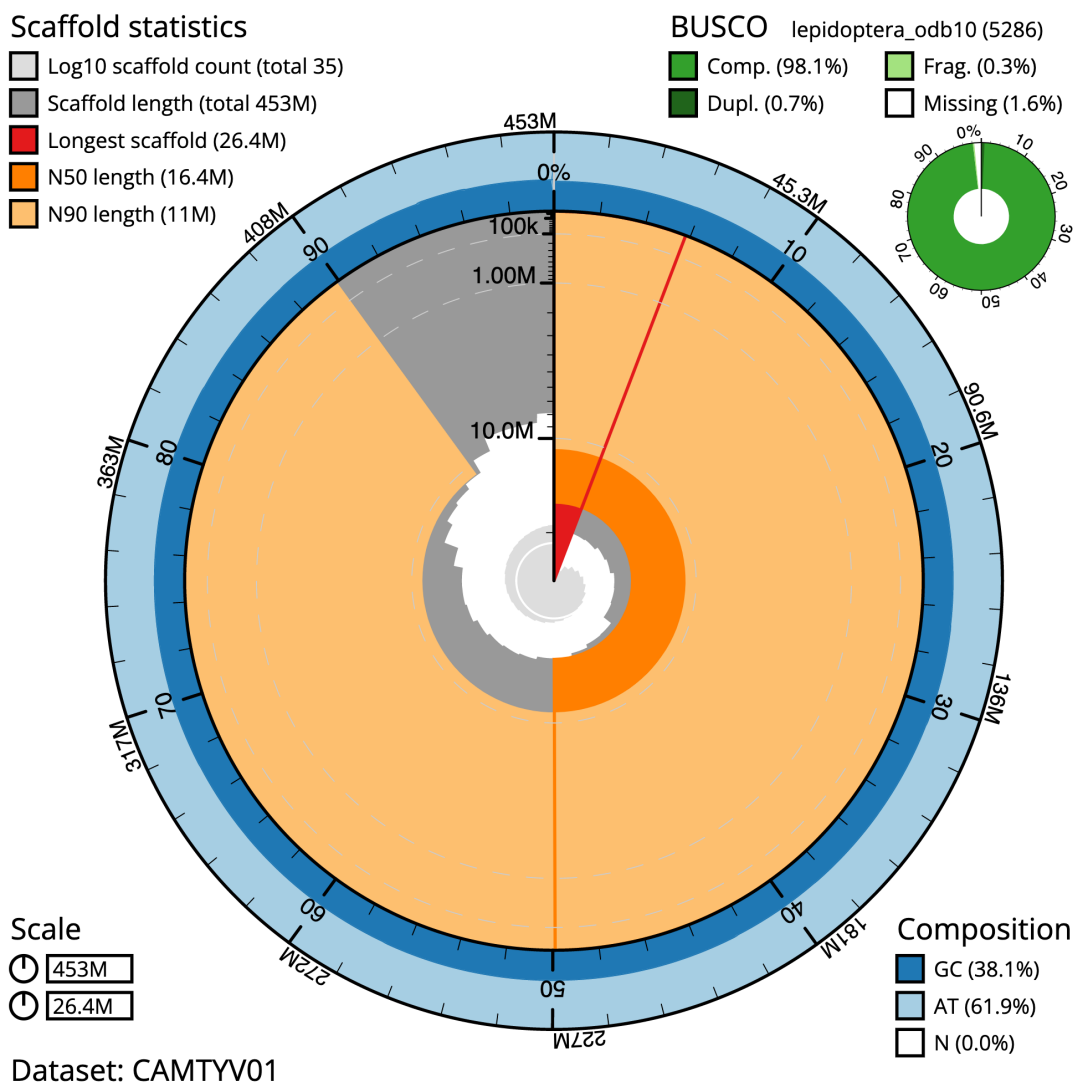
Genome assembly of *Esperia sulphurella*, ilEspSulp1.1: metrics. The BlobToolKit Snailplot shows N50 metrics and BUSCO gene completeness. The main plot is divided into 1,000 size-ordered bins around the circumference with each bin representing 0.1% of the 453,222,902 bp assembly. The distribution of scaffold lengths is shown in dark grey with the plot radius scaled to the longest scaffold present in the assembly (26,432,394 bp, shown in red). Orange and pale-orange arcs show the N50 and N90 scaffold lengths (16,376,554 and 10,956,773 bp), respectively. The pale grey spiral shows the cumulative scaffold count on a log scale with white scale lines showing successive orders of magnitude. The blue and pale-blue area around the outside of the plot shows the distribution of GC, AT and N percentages in the same bins as the inner plot. A summary of complete, fragmented, duplicated and missing BUSCO genes in the lepidoptera_odb10 set is shown in the top right. An interactive version of this figure is available at https://blobtoolkit.genomehubs.org/view/ilEspSulp1.1/dataset/CAMTYV01/snail.

The final assembly has a total length of 453.2 Mb in 35 sequence scaffolds with a scaffold N50 of 16.4 Mb ( Table 1). Most (99.97%) of the assembly sequence was assigned to 30 chromosomal-level scaffolds, representing 29 autosomes, and the Z sex chromosome. Chromosome-scale scaffolds confirmed by the Hi-C data are named in order of size ( Figure 2– Figure 5; Table 2). The assembly has a BUSCO v5.3.2 ( Manni *et al.*, 2021) completeness of 98.1% (single 97.5%, duplicated 0.7%) using the lepidoptera_odb10 reference set. While not fully phased, the assembly deposited is of one haplotype. Contigs corresponding to the second haplotype have also been deposited.

**Table 1.** Genome data for *Esperia sulphurella*, ilEspSulp1.1.

Project accession data		
Assembly identifier	ilEspSulp1.1	
Species	*Esperia sulphurella*	
Specimen	ilEspSulp1	
NCBI taxonomy ID	2870497	
BioProject	PRJEB55731	
BioSample ID	SAMEA10166811	
Isolate information	ilEspSulp1: male	
Assembly metrics *		*Benchmark*
Consensus quality (QV)	61.9	*≥ 50*
*k*-mer completeness	100%	*≥ 95%*
BUSCO **	C:98.1%[S:97.5%,D:0.7%], F:0.3%,M:1.6%,n:5,286	*C ≥ 95%*
Percentage of assembly mapped to chromosomes	99.97%	*≥ 95%*
Sex chromosomes	Z sex chromosome	*localised * *homologous * *pairs*
Organelles	Mitochondrial genome assembled	*complete * *single alleles*
Raw data accessions		
PacificBiosciences SEQUEL II	ERR10168722	
Hi-C Illumina	ERR10149550	
Genome assembly		
Assembly accession	GCA_947086405.1	
*Accession of alternate haplotype*	GCA_947086455.1	
Span (Mb)	453.2	
Number of contigs	106	
Contig N50 length (Mb)	8.2	
Number of scaffolds	35	
Scaffold N50 length (Mb)	16.4	
Longest scaffold (Mb)	26.4	

* Assembly metric benchmarks are adapted from column VGP-2020 of “Table 1: Proposed standards and metrics for defining genome assembly quality” from ( Rhie *et al.*, 2021). ** BUSCO scores based on the lepidoptera_odb10 BUSCO set using v5.3.2. C = complete [S = single copy, D = duplicated], F = fragmented, M = missing, n = number of orthologues in comparison. A full set of BUSCO scores is available at https://blobtoolkit.genomehubs.org/view/ilEspSulp1.1/dataset/CAMTYV01/busco.

**Figure 3.**
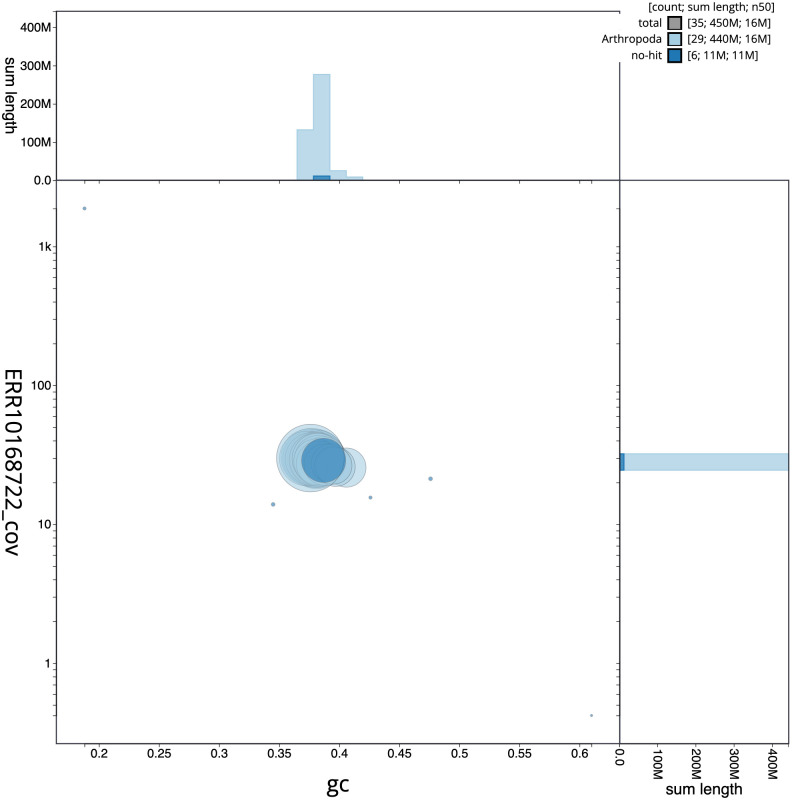
Genome assembly of *Esperia sulphurella*, ilEspSulp1.1: GC coverage. BlobToolKit GC-coverage plot. Scaffolds are coloured by phylum. Circles are sized in proportion to scaffold length. Histograms show the distribution of scaffold length sum along each axis. An interactive version of this figure is available at https://blobtoolkit.genomehubs.org/view/ilEspSulp1.1/dataset/CAMTYV01/blob.

**Figure 4.**
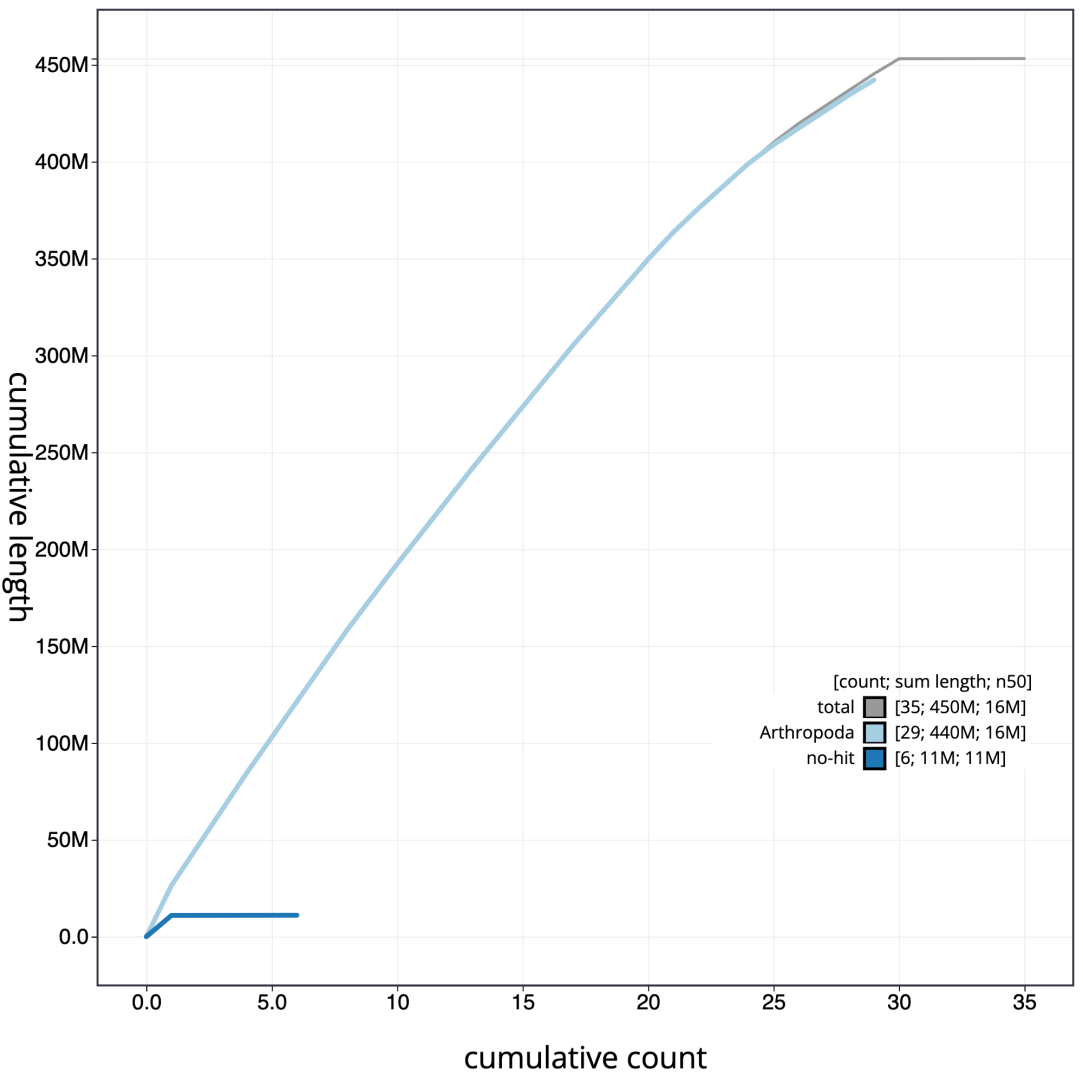
Genome assembly of *Esperia sulphurella*, ilEspSulp1.1: cumulative sequence. BlobToolKit cumulative sequence plot. The grey line shows cumulative length for all scaffolds. Coloured lines show cumulative lengths of scaffolds assigned to each phylum using the buscogenes taxrule. An interactive version of this figure is available at https://blobtoolkit.genomehubs.org/view/ilEspSulp1.1/dataset/CAMTYV01/cumulative.

**Figure 5.**
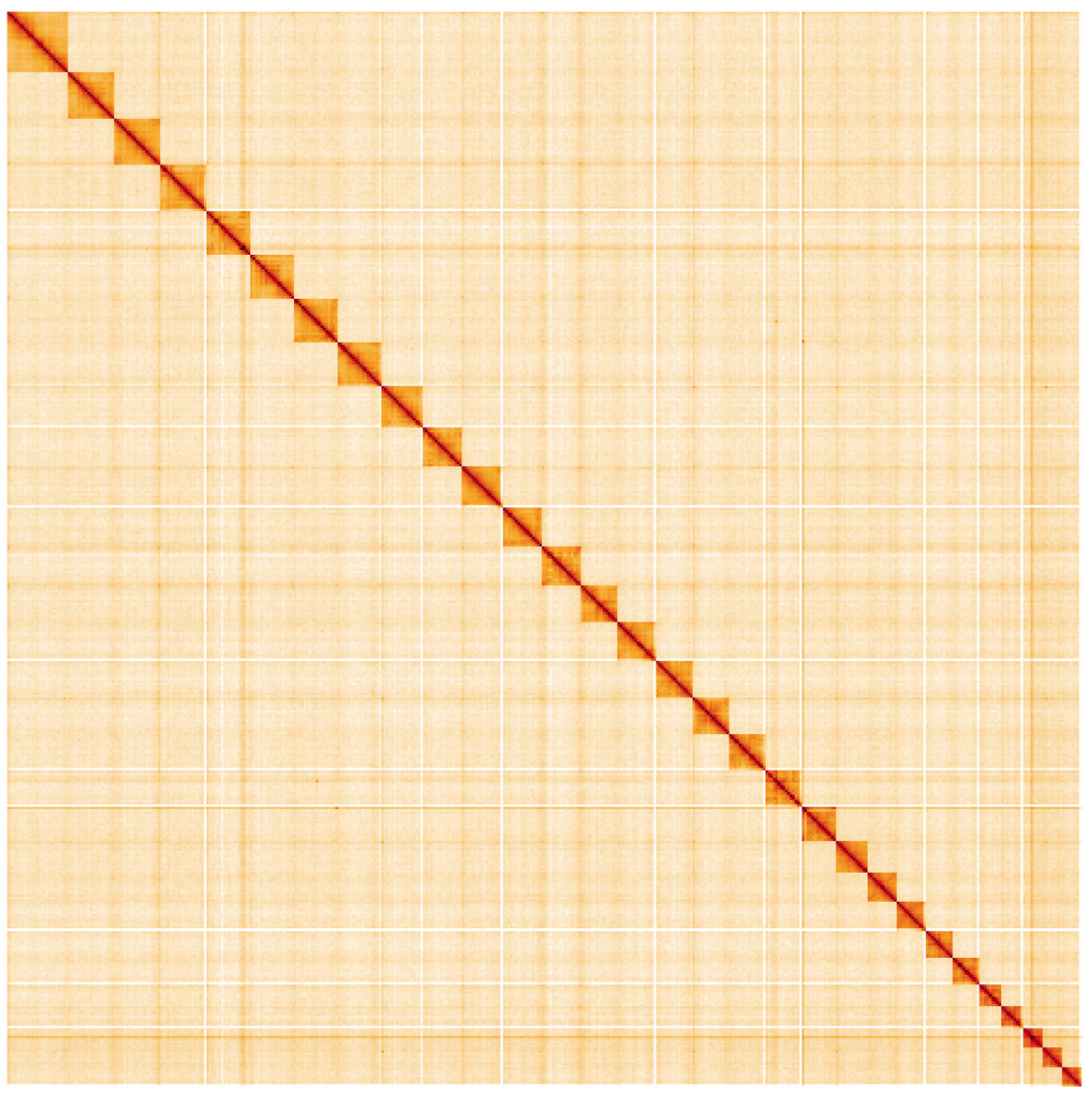
Genome assembly of *Esperia sulphurella*, ilEspSulp1.1: Hi-C contact map. Hi-C contact map of the ilEspSulp1.1 assembly, visualised using HiGlass. Chromosomes are shown in order of size from left to right and top to bottom. An interactive version of this figure may be viewed at https://genome-note-higlass.tol.sanger.ac.uk/l/?d=b_dgwdvFRF-QNCmAPPStBw.

**Table 2.** Chromosomal pseudomolecules in the genome assembly of *Esperia sulphurella*, ilEspSulp1.

INSDC accession	Chromosome	Size (Mb)	GC%
OX352258.1	1	19.56	38
OX352259.1	2	19.45	38.1
OX352260.1	3	19.05	38.1
OX352261.1	4	18.82	37.7
OX352262.1	5	18.37	37.6
OX352263.1	6	18.32	38.1
OX352264.1	7	18.22	37.5
OX352265.1	8	17.12	37.6
OX352266.1	9	17.07	37.3
OX352267.1	10	16.72	38
OX352268.1	11	16.38	37.9
OX352269.1	12	16.38	38.1
OX352270.1	13	16.06	38.2
OX352271.1	14	15.91	37.7
OX352272.1	15	15.77	38.1
OX352273.1	16	15.44	38
OX352274.1	17	14.97	38.4
OX352275.1	18	14.8	38.2
OX352276.1	19	14.78	38.2
OX352277.1	20	13.83	38.3
OX352278.1	21	12.34	38.7
OX352279.1	22	11.74	38.1
OX352280.1	23	11.49	38.3
OX352281.1	24	10.96	38.7
OX352282.1	25	9.71	38.8
OX352283.1	26	8.55	39.3
OX352284.1	27	8.55	40.6
OX352285.1	28	8.52	39.7
OX352286.1	29	7.81	39.5
OX352287.1	Z	26.43	37.6
OX352288.1	MT	0.02	19.1

## Methods

### Sample acquisition and nucleic acid extraction

A male *Esperia sulphurella* (specimen ToLID ilEspSulp1, specimen Ox001336) was collected in Wallingford, Oxfordshire (biological vice-county: Berkshire) (latitude 51.60, longitude –1.14) on 14 May 2021. The specimen was taken from a garden habitat by Peter Holland (University of Oxford) using a light trap. The specimen was identified as a male on the basis of wing markings by Peter Holland, and was frozen at –80°C.

DNA was extracted at the Tree of Life laboratory, Wellcome Sanger Institute (WSI). The ilEspSulp1 sample was weighed and dissected on dry ice with tissue set aside for Hi-C sequencing. Whole organism tissue was disrupted using a Nippi Powermasher fitted with a BioMasher pestle. High molecular weight (HMW) DNA was extracted using the Qiagen MagAttract HMW DNA extraction kit. HMW DNA was sheared into an average fragment size of 12–20 kb in a Megaruptor 3 system with speed setting 30. Sheared DNA was purified by solid-phase reversible immobilisation using AMPure PB beads with a 1.8X ratio of beads to sample to remove the shorter fragments and concentrate the DNA sample. The concentration of the sheared and purified DNA was assessed using a Nanodrop spectrophotometer and Qubit Fluorometer and Qubit dsDNA High Sensitivity Assay kit. Fragment size distribution was evaluated by running the sample on the FemtoPulse system.

### Sequencing

Pacific Biosciences HiFi circular consensus DNA sequencing libraries were constructed according to the manufacturers’ instructions. DNA sequencing was performed by the Scientific Operations core at the WSI on Pacific Biosciences SEQUEL II (HiFi) instrument. Hi-C data were also generated from tissue of ilEspSulp1 using the Arima v2 kit and sequenced on the Illumina NovaSeq 6000 instrument.

### Genome assembly

Assembly was carried out with Hifiasm ( Cheng *et al.*, 2021) and haplotypic duplication was identified and removed with purge_dups ( Guan *et al.*, 2020). One round of polishing was performed by aligning 10X Genomics read data to the assembly with Long Ranger ALIGN, calling variants with FreeBayes ( Garrison & Marth, 2012). The assembly was then scaffolded with Hi-C data ( Rao *et al.*, 2014) using YaHS ( Zhou *et al.,* 2023). The assembly was checked for contamination and corrected using the gEVAL system ( Chow *et al.*, 2016) as described previously ( Howe *et al.*, 2021). Manual curation was performed using gEVAL, HiGlass ( Kerpedjiev *et al.*, 2018) and Pretext ( Harry, 2022). The mitochondrial genome was assembled using MitoHiFi ( Uliano-Silva *et al.*, 2022), which performed annotation using MitoFinder ( Allio *et al.*, 2020). The genome was analysed and BUSCO scores generated within the BlobToolKit environment ( Challis *et al.*, 2020). Table 3 contains a list of all software tool versions used, where appropriate.

**Table 3.** Software tools and versions used.

Software tool	Version	Source
BlobToolKit	4.0.7	Challis *et al.*, 2020
gEVAL	N/A	Chow *et al.*, 2016
Hifiasm	0.16.1-r375	Cheng *et al.*, 2021
HiGlass	1.11.6	Kerpedjiev *et al.*, 2018
MitoHiFi	2	Uliano-Silva *et al.*, 2022
PretextView	0.2	Harry, 2022
purge_dups	1.2.3	Guan *et al.*, 2020
YaHS	yahs-1.1.91eebc2	Zhou *et al.*, 2023

### Ethics and compliance issues

The materials that have contributed to this genome note have been supplied by a Darwin Tree of Life Partner. The submission of materials by a Darwin Tree of Life Partner is subject to the Darwin Tree of Life Project Sampling Code of Practice. By agreeing with and signing up to the Sampling Code of Practice, the Darwin Tree of Life Partner agrees they will meet the legal and ethical requirements and standards set out within this document in respect of all samples acquired for, and supplied to, the Darwin Tree of Life Project. All efforts are undertaken to minimise the suffering of animals used for sequencing. Each transfer of samples is further undertaken according to a Research Collaboration Agreement or Material Transfer Agreement entered into by the Darwin Tree of Life Partner, Genome Research Limited (operating as the Wellcome Sanger Institute), and in some circumstances other Darwin Tree of Life collaborators.

## Data Availability

European Nucleotide Archive: *Esperia sulphurella* (sulphur tubic). Accession number PRJEB55731; https://identifiers.org/ena.embl/PRJEB55731 ( Wellcome Sanger Institute, 2022) The genome sequence is released openly for reuse. The *Esperia sulphurella* genome sequencing initiative is part of the Darwin Tree of Life (DToL) project. All raw sequence data and the assembly have been deposited in INSDC databases. The genome will be annotated using available RNA-Seq data and presented through the Ensembl pipeline at the European Bioinformatics Institute. Raw data and assembly accession identifiers are reported in Table 1.

## Author information

Members of the University of Oxford and Wytham Woods Genome Acquisition Lab are listed here: https://doi.org/10.5281/zenodo.4789928.

Members of the Darwin Tree of Life Barcoding collective are listed here: https://doi.org/10.5281/zenodo.4893703.

Members of the Wellcome Sanger Institute Tree of Life programme are listed here: https://doi.org/10.5281/zenodo.4783585.

Members of Wellcome Sanger Institute Scientific Operations: DNA Pipelines collective are listed here: https://doi.org/10.5281/zenodo.4790455 .

Members of the Tree of Life Core Informatics collective are listed here: https://doi.org/10.5281/zenodo.5013541.

Members of the Darwin Tree of Life Consortium are listed here: https://doi.org/10.5281/zenodo.4783558.
