# Supplementary material for: The genome sequence of the Sulphur Tubic, Esperia sulphurella (Fabricius, 1775)
Source: Wellcome Open Res. 2023 Apr 6;8:156. [Version 1] doi: 10.12688/wellcomeopenres.19212.1 (PMC10690030; doi:10.12688/wellcomeopenres.19212.1)
Supplement: Peer review history

# Reviewer report: Reviewer response for version 1

**DOI:** 10.21956/wellcomeopenres.21294.r70226

**Published:** 2023-12-15

**Authors:** Alexander Kneubehl

**Affiliations:** 1 Department of Pediatrics, Baylor College of Medicine, Houston, TX, USA

The work presented here is a description of the assembly of a moth ( *Esperia sulphurella*). Holland provides necessary metadata regarding sample collection, speciation, and sex of the sample. Multiple sequencing modalities are used (PacBio HiFi and Hi-C sequencing) for the assembly, polishing, and scaffolding. 10x Genomics data is used for polishing but the generation of these data is not indicated in the methods nor is a sequence repository accession number provided for these data anywhere in the Data Note. Otherwise, methods and associated code metadata are acceptable.

Are sufficient details of methods and materials provided to allow replication by others?

Yes

Is the rationale for creating the dataset(s) clearly described?

Yes

Are the datasets clearly presented in a useable and accessible format?

Partly

Are the protocols appropriate and is the work technically sound?

Yes

Reviewer Expertise:

Genomics, vector biology, virology, bacteriology

I confirm that I have read this submission and believe that I have an appropriate level of expertise to confirm that it is of an acceptable scientific standard, however I have significant reservations, as outlined above.

# Reviewer report: Reviewer response for version 1

**DOI:** 10.21956/wellcomeopenres.21294.r70224

**Published:** 2023-11-30

**Authors:** Zachary Cohen

**Affiliations:** 1 Insect Control and Cotton Disease Research Unit, Southern Plains Agricultural Research Center,Agricultural Research Service, United States Department of Agriculture, College Station, TX, USA

This report is for a reference quality genome assembly of the Sulphur Tubic moth with sound methodology and presentation of final results. The authors have successfully generated the complete assembly with 29 autosomes, the Z sex chromosome and mitochondria. I look forward to tracking this assembly as the official gene set is produced.

Are sufficient details of methods and materials provided to allow replication by others?

Yes

Is the rationale for creating the dataset(s) clearly described?

Yes

Are the datasets clearly presented in a useable and accessible format?

Yes

Are the protocols appropriate and is the work technically sound?

Yes

Reviewer Expertise:

entomology, computational biology, evolution

I confirm that I have read this submission and believe that I have an appropriate level of expertise to confirm that it is of an acceptable scientific standard.

# Reviewer report: Reviewer response for version 1

**DOI:** 10.21956/wellcomeopenres.21294.r56630

**Published:** 2023-05-02

**Authors:** Maurijn van der Zee

**Affiliations:** 1 Institute of Biology, Leiden University, Sylviusweg, Leiden, The Netherlands

Peter Holland provides the genome sequence of the Sulphur Tubic moth. Judging from the description, the geome is of high quality, as the 35 scaffolds nearly represent the number of chromosomes (30), and the BUSCO score practically suggest completion. The manual curation particularly improved the assembly. Thus, this will be a valubale contribution to the community. Note that I could download the genomic and transcriptomic fasq files from the site, but not the actual assembly (or did I overlook something?). I trust that this will be made easily available.

Are sufficient details of methods and materials provided to allow replication by others?

Yes

Is the rationale for creating the dataset(s) clearly described?

Yes

Are the datasets clearly presented in a useable and accessible format?

No

Are the protocols appropriate and is the work technically sound?

Yes

Reviewer Expertise:

evo-devo, insects, comparative genomics

I confirm that I have read this submission and believe that I have an appropriate level of expertise to confirm that it is of an acceptable scientific standard.
